# Supplementary material for: A dendritic hexamer acceptor enables 19.4% efficiency with exceptional stability in organic solar cells
Source: Nat Commun. 2025 Jan 20;16:871. doi: 10.1038/s41467-025-56225-x (PMC11747272; doi:10.1038/s41467-025-56225-x)
Supplement: Supplementary file 1 — Supplementary Information [file 41467_2025_56225_MOESM1_ESM.pdf]

Supplementary Information

A Dendritic Hexamer Acceptor Enables 19.4% Efficiency with Exceptional Stability in Organic Solar Cells

Tao Jia [1,2,†], Tao Lin [1,†],Yang Yang[1,†], Lunbi Wu [1], Huimin Cai [1], Zesheng Zhang [3], Kangfeng Lin [1], Yulong Hai [4], Yongmin Luo [4], Ruijie Ma [5]✉, Yao Li [4], Top Archie Dela Peña [4], Sha Liu [6], Jie Zhang [3], Chunchen Liu [3], Junwu Chen [3], Jiaying Wu [4]✉, Shengjian Liu [2]✉ & Fei Huang[3]

[1]School of Optoelectronic Engineering, Guangdong Polytechnic Normal University, Guangzhou, 510665, China

[2]School of Chemistry, Guangzhou Key Laboratory of Materials for Energy Conversion and Storage, Key Laboratory of Electronic Chemicals for Integrated Circuit Packaging, South China Normal University (SCNU), Guangzhou 510006, China

[3]Institute of Polymer Optoelectronic Materials and Devices, State Key Laboratory of Luminescent Materials and Devices, South China University of Technology, Guangzhou 510640, China

[4]Advanced Materials Thrust, Function Hub, The Hong Kong University of Science and Technology (Guangzhou), Nansha 511400, Guangzhou, China

[5]Department of Electrical and Electronic Engineering, Research Institute for Smart Energy (RISE), Photonic Research Institute (PRI), The Hong Kong Polytechnic University, Hong Kong, China

[6]Dongguan Key Laboratory of Interdisciplinary Science for Advanced Materials and Large-Scale Scientific Facilities, School of Physical Sciences, Great Bay University, Dongguan, Guangdong, 523000, P. R. China

[†]These authors contributed equally: Tao Jia, Tao Lin, Yang Yang.

✉e-mail: ruijie.ma@polyu.edu.hk; jiayingwu@ust.hk; shengjian.liu@m.scnu.edu.cn

Supplementary Methods

Materials synthesis

12-(8-Bromooctyl)-13-(2-decyltetradecyl)-3,9-diundecyl-12,13-dihydro-[1,2,5]thiadiazolo[3,4-e]thieno[2'',3'':4',5']thieno[2',3':4,5]pyrrolo[3,2-g]thieno[2',3':4,5]thieno[3,2-b]indole (**compound 2**)

Compound **1** (7.47 g, 10 mmol), 11-(bromomethyl)tricosane (3.97 g, 9.5 mmol), KI (332 mg, 2 mmol), K_2CO_3 (2.76 g, 20 mmol), and DMF (100 mL) were added into a round-bottom flask under air atmosphere. The reaction was stirred at 110 °C for 18 hours. Then, 1,8-dibromooctane (13.6 g, 50 mmol) was added into the flask and the reaction mixture was stirred at 110 °C for another 12 h. After cooling to room temperature, the reaction was extracted with dichloromethane. The organic layers were combined and washed with saturated brine for three times. After evaporating the solvent, the residue was purified by column chromatography with petroleum ether (PE)/dichloromethane (DCM) (*v*:*v*, 3/1) as eluent to afford compound **2** as an orange solid (10.46 g, yield 82%).

2,3,7,8,12,13-Hexakis((8-(13-(2-decyltetradecyl)-3,9-diundecyl-[1,2,5]thiadiazolo[3,4-e]thieno[2'',3'':4',5']thieno[2',3':4,5]pyrrolo[3,2-g]thieno[2',3':4,5]thieno[3,2-b]indol-12(13H)-yl)octyl)oxy)-10,15-dihydro-5H-tribenzo[a,d,g][9]annulene (**Six-H**)

Compound 10,15-Dihydro-5H-tribenzo[a,d,g][9]annulene-2,3,7,8,12,13-hexaol (100mg, 0.273 mmol), K_2CO_3 (678 mg, 4.914 mmol), and ultra-dry DMF solvent (30 mL) were added into a two-neck flask under argon atmosphere. The reaction mixture was stirred at 110 °C under argon atmosphere for 30 min. Then compound 2 (3.133g, 2.457 mmol) were added into the flask. The reaction mixture was then stirred at 90 °C for another 72 hours. Potassium carbonate (300 mg) was added to the reaction system every 24 hours throughout the reaction. After cooling to room temperature, the deionized water was added into the flask. The crude product was extracted with dichloromethane. The organic layers were combined and washed with saturated brine for three times. After evaporating the solvent, the residue was purified by column chromatography with PE/DCM (*v*:*v*, 3/1~2/1) as eluent to afford compound Six-H as

an orange solid (1.748 g, yield 86%). The superfluous reactive material compound 2 can be recovered after column chromatography. ^{1}H NMR (600 MHz, $CDCl_3$) δ 6.91 (s, 6H), 6.84 (s, 6H), 6.63 (s, 6H), 4.54 – 4.44 (m, 30H), 3.72 – 3.64 (m, 16H),2.72 (dt, *J* = 15.4, 7.8 Hz, 30H), 2.06 (m, 6H), 1.78 (tt, *J* = 15.9, 7.9 Hz, 32H), 1.67 (m, 12H), 1.30 – 1.21 (m, 226H), 1.07 – 0.90 (m, 160H), 0.90 – 0.81 (m, 160H), 0.76 – 0.71 (m, 36H). ^{13}C NMR (151 MHz, $CDCl_3$) δ 147.69, 147.55, 147.51, 142.12, 142.05, 137.12, 136.92, 136.81, 136.71, 132.12, 131.45, 130.97, 123.58, 123.22, 122.82, 122.75, 119.19, 119.08, 116.11, 111.83, 111.41, 69.34, 54.73, 50.95, 38.64, 31.96, 31.95, 31.92, 30.45, 30.26, 29.73, 29.68, 29.60, 29.55, 29.50, 29.39, 29.30, 29.20, 29.05, 28.93, 28.82, 26.38, 25.83, 25.77, 25.43, 22.71, 14.14. MALDI-TOF m/z calcd. for $C_{453}H_{690}N_{24}O_6S_{30}$, 7530.5; found, 7529.2.

13,13',13'',13''',13'''',13'''''-(((10,15-Dihydro-5H-tribenzo[a,d,g][9]annulene-2,3,7,8,12,13-hexayl)hexakis(oxy))hexakis(octane-8,1-diyl))hexakis(12-(2-decyltetradecyl)-3,9-diundecyl-12,13-dihydro-[1,2,5]thiadiazolo[3,4-e]thieno[2'',3'':4',5']thieno[2',3':4,5]pyrrolo[3,2-g]thieno[2',3':4,5]thieno[3,2-b]indole-2,10-dicarbaldehyde) (**Six-CHO**)

To a a two-neck flask containing dried DMF (50 mL), $POCl_3$ (1 mL) was slowly added under argon atmosphere. After stirring at room temperature for 30 min, a solution of Six-H (753 mg, 0.1 mmol) in dried DCM (10 mL) was added to the flask. The reaction mixture was then stirred at 100 °C for another 1 hour. After cooled to room temperature, the reaction mixture was slowly poured into Na_2CO_3 aqueous solution. The product was extracted with DCM. After evaporating the organic solvent by reduced pressure, the residue was purified by column chromatography on silica gel using PE/DCM (*v*/*v*, 1:1.5) as eluent to afford **Six-CHO** as yellow viscous solid (724 mg, yield 92%). ^{1}H NMR (600 MHz, $CDCl_3$) δ 10.03 (s, 6H), 9.98 (s, 6H), 6.65 (s, 6H), 4.61 – 4.48 (m, 30H), 3.70 (t, *J* = 6.4 Hz, 12H), 3.04 (dt, *J* = 13.2, 7.8 Hz, 24H), 2.07 – 1.98 (m, 6H), 1.83 (m, 32H), 1.29 – 1.17 (m, 230H), 1.11 (m, 100H), 1.00 (m, 52H), 0.95 – 0.90 (m, 48H), 0.88 – 0.81 (m, 122H), 0.72 (m, 16H). ^{13}C NMR (151 MHz, $CDCl_3$) δ 181.44, 181.40, 181.30, 147.76, 147.41, 147.27, 147.18, 146.67, 146.61, 146.56, 143.12, 143.05, 142.99, 142.84, 142.47, 142.37, 137.82, 137.63,

136.92, 136.82, 136.79, 136.63, 136.55, 136.27, 135.73, 133.59, 133.07, 132.68, 132.23, 132.20, 132.07, 132.01, 129.71, 129.47, 129.19, 128.99, 127.57, 127.35, 123.40, 123.03, 122.66, 119.90, 116.22, 112.43, 112.19, 110.82, 69.42, 54.99, 54.83, 51.11, 38.97, 38.84, 36.41, 31.93, 31.92, 31.88, 30.89, 30.38, 30.35, 30.27, 29.71, 29.67, 29.64, 29.57, 29.51, 29.47, 29.36, 29.32, 29.26, 29.20, 29.11, 28.81, 28.12, 28.04, 26.57, 25.91, 25.45, 22.70, 22.67, 14.13. MALDI-TOF m/z calcd. for $C_{465}H_{690}N_{24}O_{18}S_{30}$, 7866.6; found, 7865.3.

Six-IC：

To a two-neck flask containing Six-CHO (393 mg, 0.05 mmol) and 2-(5,6-difluoro-3-oxo-2,3-dihydro-1*H*-inden-1-ylidene) malononitrile (345.3 mg, 1.5 mmol), chloroform (30 ml) and pyridine (1 mL) were added under argon atmosphere. The reaction mixture was stirred at 65 °C overnight. After evaporating the organic solvent, the residue was purified by column chromatography using PE/DCM (*v*/*v*, 1:1.2) to give the target product **Six-IC** as a dark flake solid (468.5mg, yield 90%). ^{1}H NMR (500 MHz, $C_2D_2Cl_4$) δ 8.53 – 8.29 (m, 24 H), 7.49 (m, 12H), 6.87 (m, 6H), 4.71 (m, 30H), 4.07 (m, 12H), 2.81 (m, 30H), 1.97– 1.58 (m, 118H), 1.47 – 0.89 (m, 362H), 0.84 – 0.58 (m, 120H). ^{13}C NMR (126 MHz,) δ 185.49, 185.33, 157.84, 157.79, 157.55, 155.34, 155.26, 155.14, 153.84, 153.80, 153.55, 153.30, 153.16, 153.05, 148.81, 148.59, 147.39, 147.38, 147.05, 145.43, 145.40, 145.20, 137.17, 137.01, 136.62, 136.56, 135.03, 134.59, 134.45, 134.40, 134.11, 133.93, 133.23, 133.04, 132.82, 132.77, 132.62, 132.57, 131.40, 130.87, 130.80, 119.69, 114.73, 114.57, 113.75, 113.07, 112.02, 70.34, 69.23, 55.57, 51.80, 39.67, 31.98, 31.89, 31.82, 31.42, 31.35, 30.85, 30.52, 30.35, 29.93, 29.68, 29.58, 29.51, 29.38, 29.26, 29.19, 27.81, 26.94, 26.25, 26.19, 22.70, 22.62, 22.58, 14.07, 13.99, 13.96. MALDI-TOF m/z calcd. for $C_{609}H_{714}F_{24}N_{48}O_{18}S_{30}$, 10412.5; found, 10411.5.

Solution NMR and Mass Spectra Measurements

The ^{1}H NMR and ^{13}C NMR spectra were measured on a Bruker Avance III HD (500 MHz) or Bruker AVANCE NEO (600 MHz) spectrometer with tetramethylsilane (TMS) as the internal reference at room temperature or 100 °C. Mass spectra were

measured on Bruker ultrafleXtreme instrument.

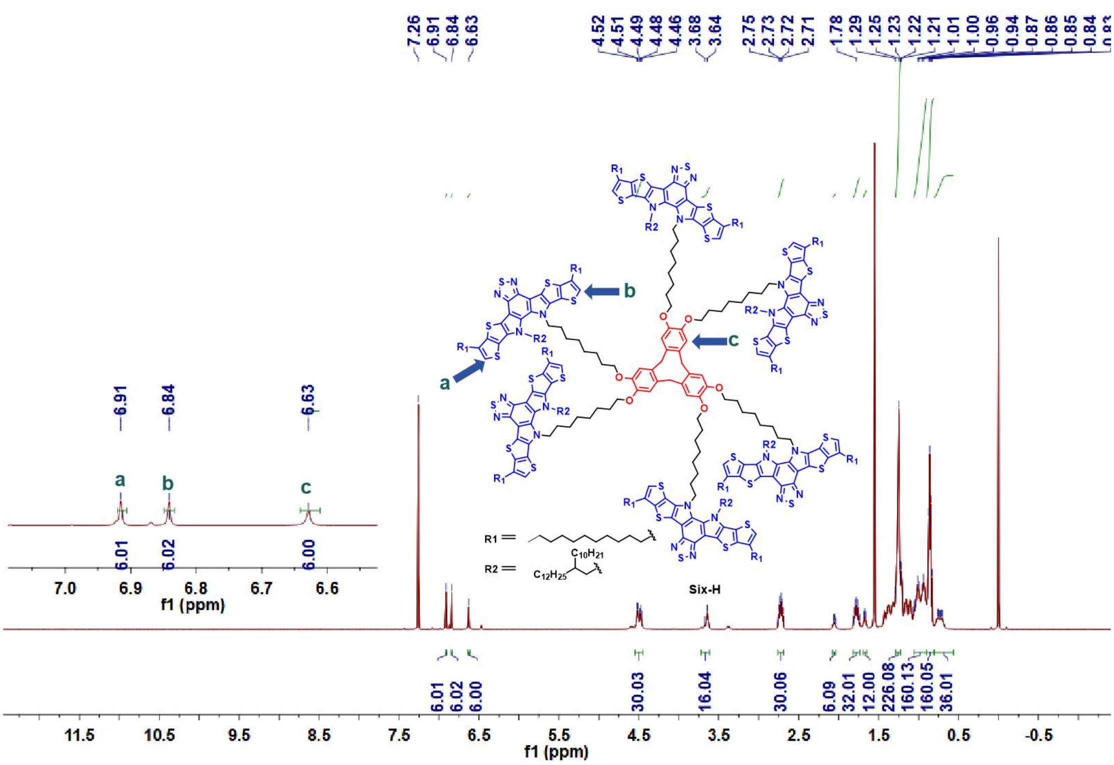

Supplementary Fig. 1. ^{1}H NMR spectrum of **Six-H** in $CDCl_3$.

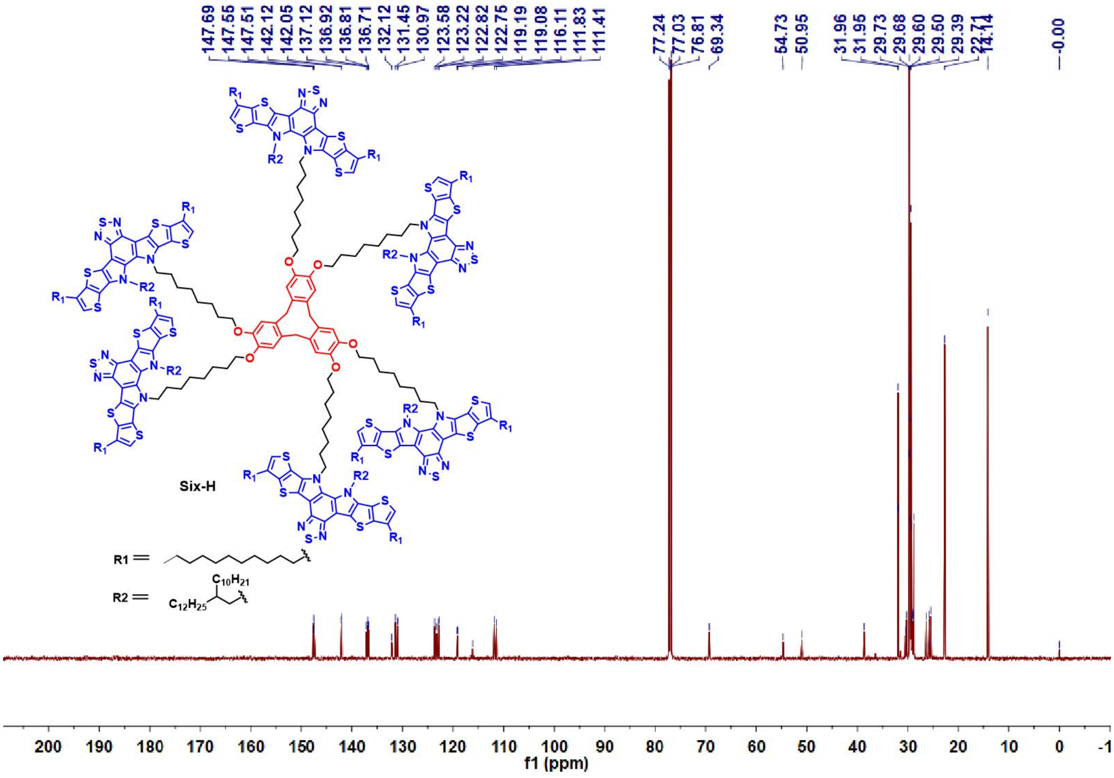

Supplementary Fig. 2. ^{13}C NMR spectrum of **Six-H** in $CDCl_3$.

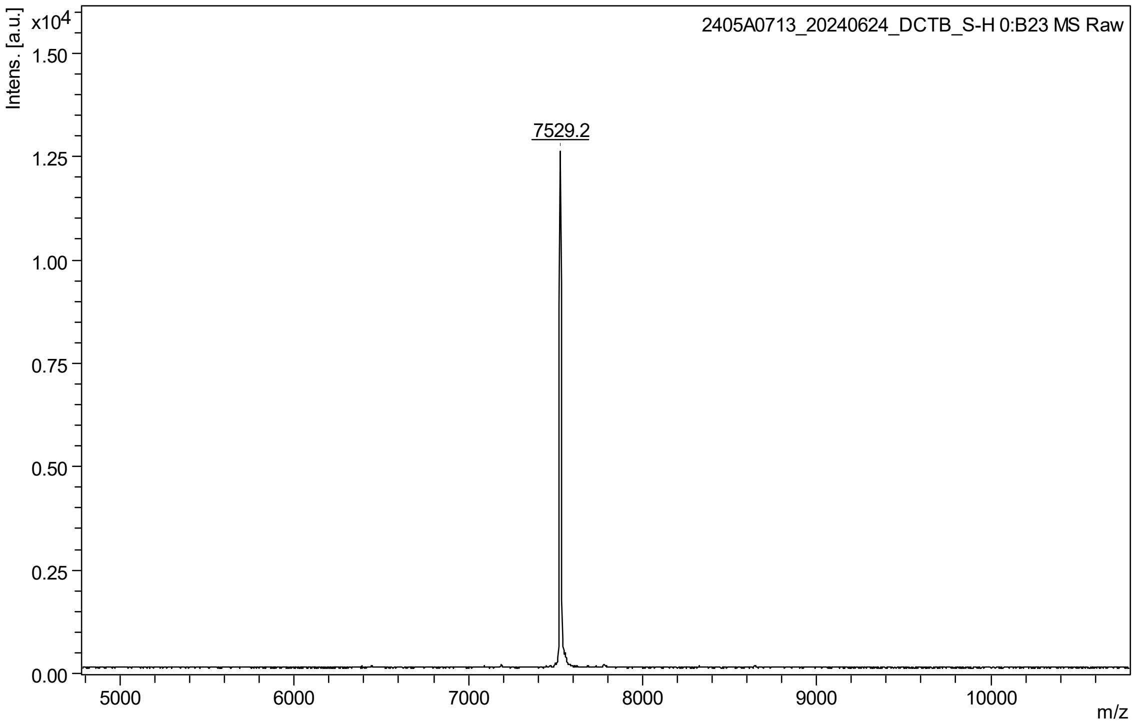

Supplementary Fig. 3. The mass spectrum of **Six-H**.

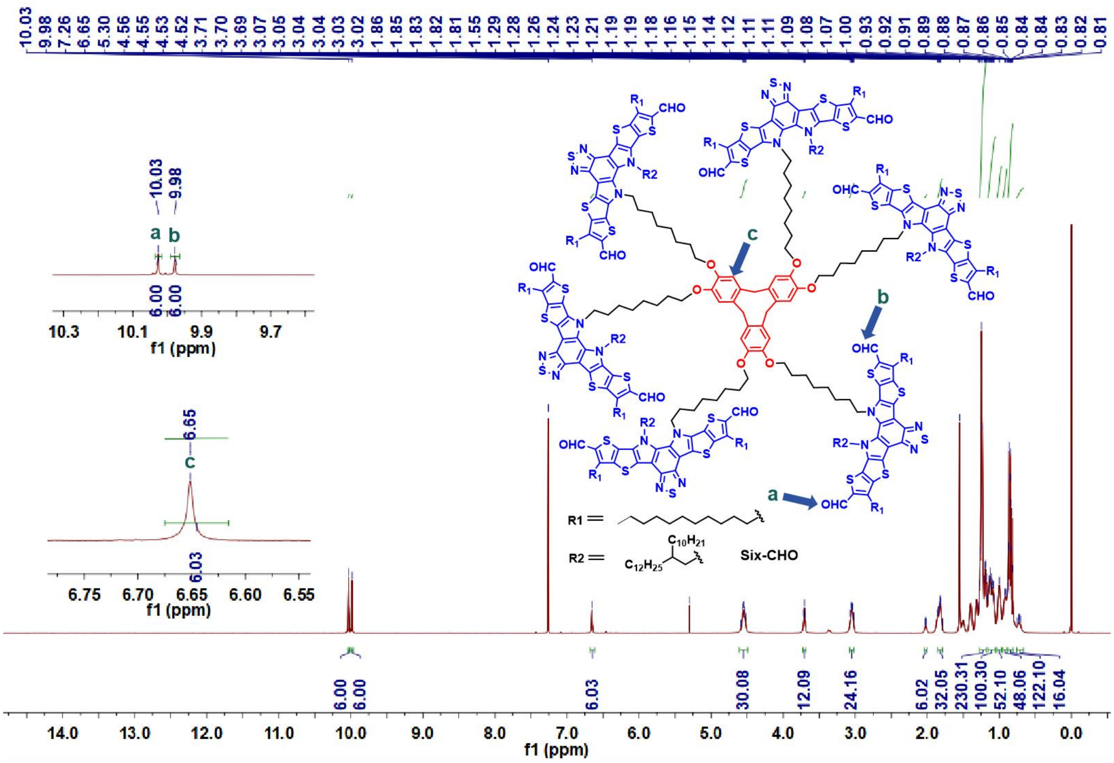

Supplementary Fig. 4. ^{1}H NMR spectrum of **Six-CHO** in $CDCl_3$.

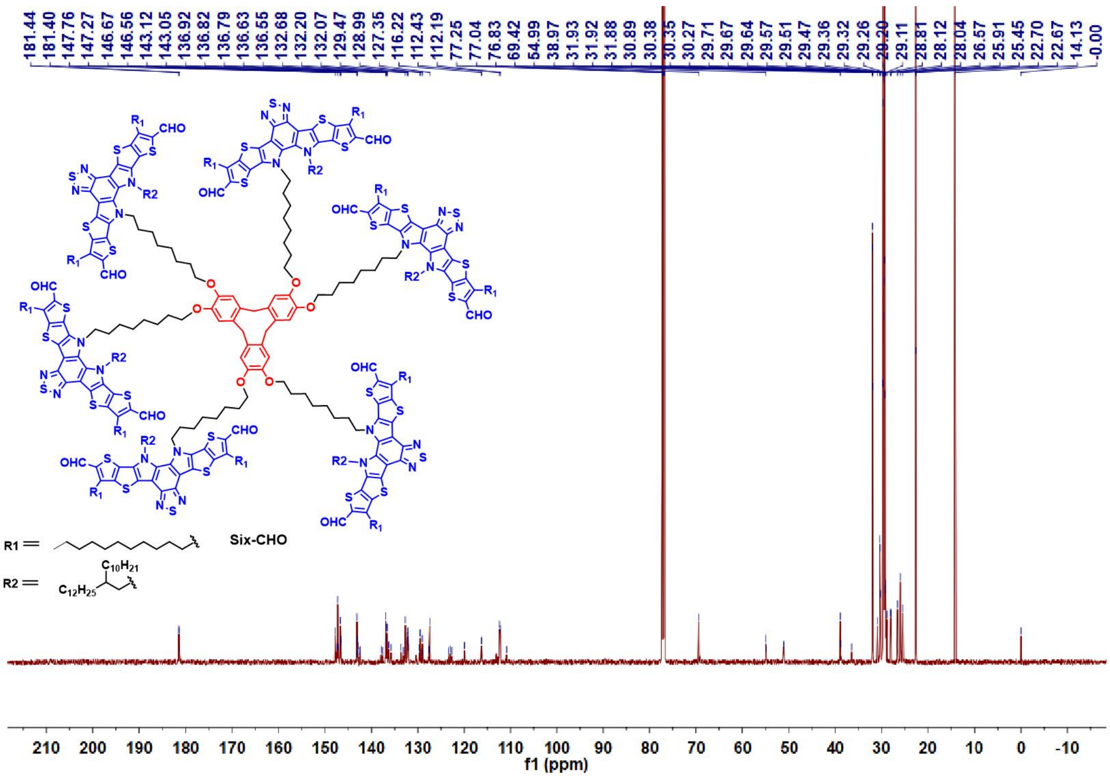

Supplementary Fig. 5. ^{13}C NMR spectrum of **Six-CHO** in $CDCl_3$.

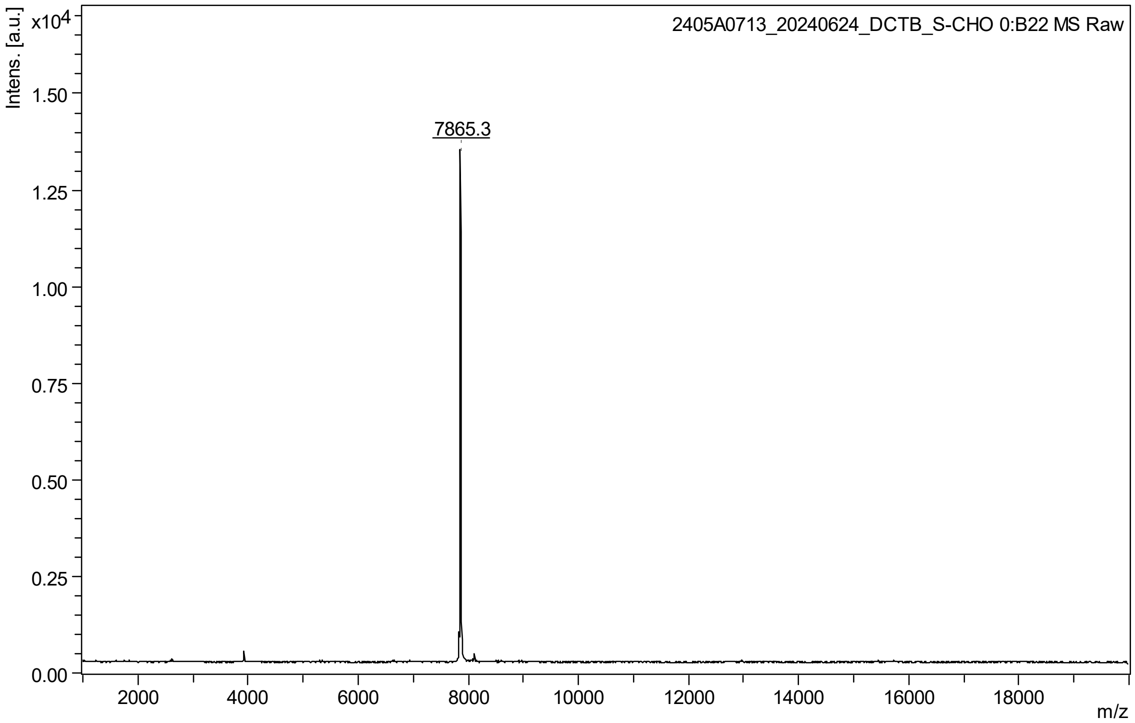

Supplementary Fig. 6. The mass spectrum of **Six-CHO**.

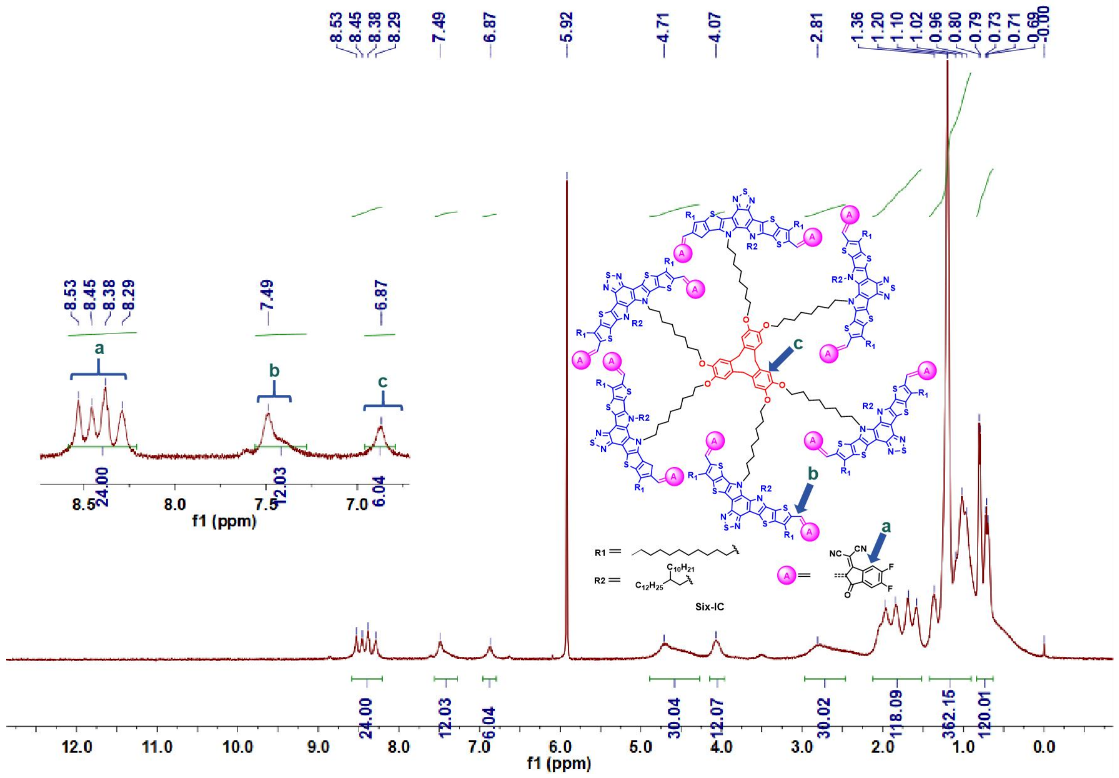

Supplementary Fig. 7. ^{1}H NMR spectrum of **Six-IC** in $C_2D_2Cl_4$.

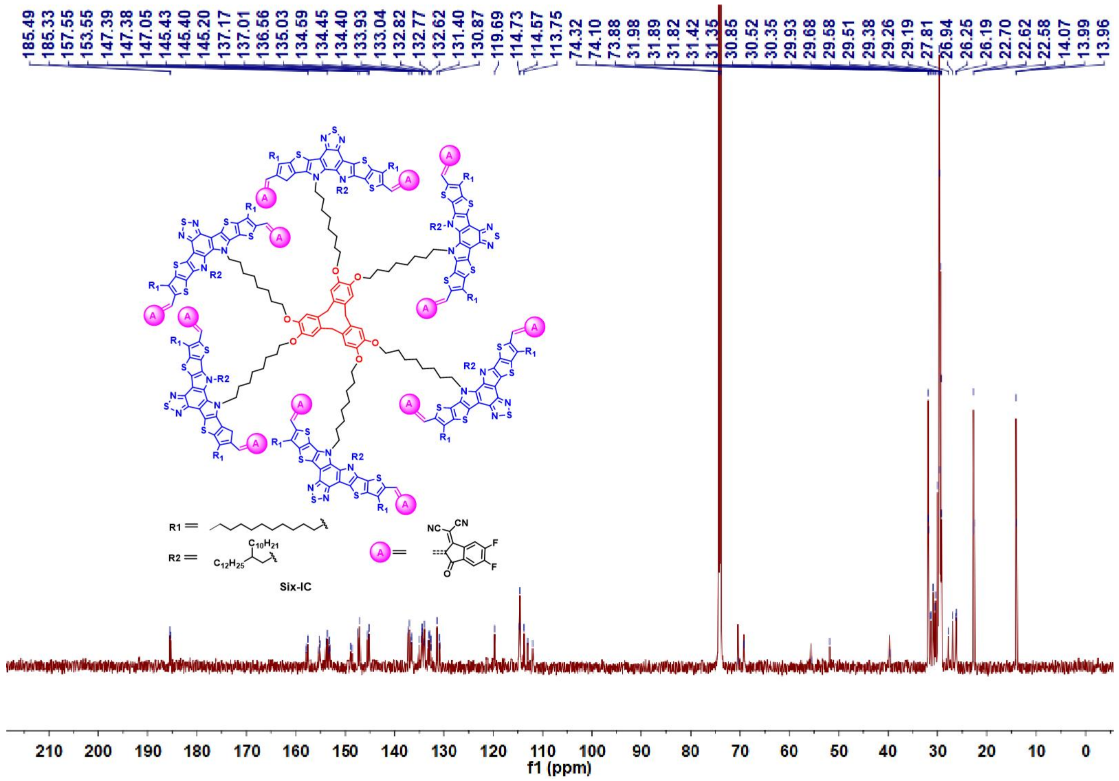

Supplementary Fig. 8. ^{13}C NMR spectrum of **Six-IC** in $C_2D_2Cl_4$ at 100 ^{o}C.

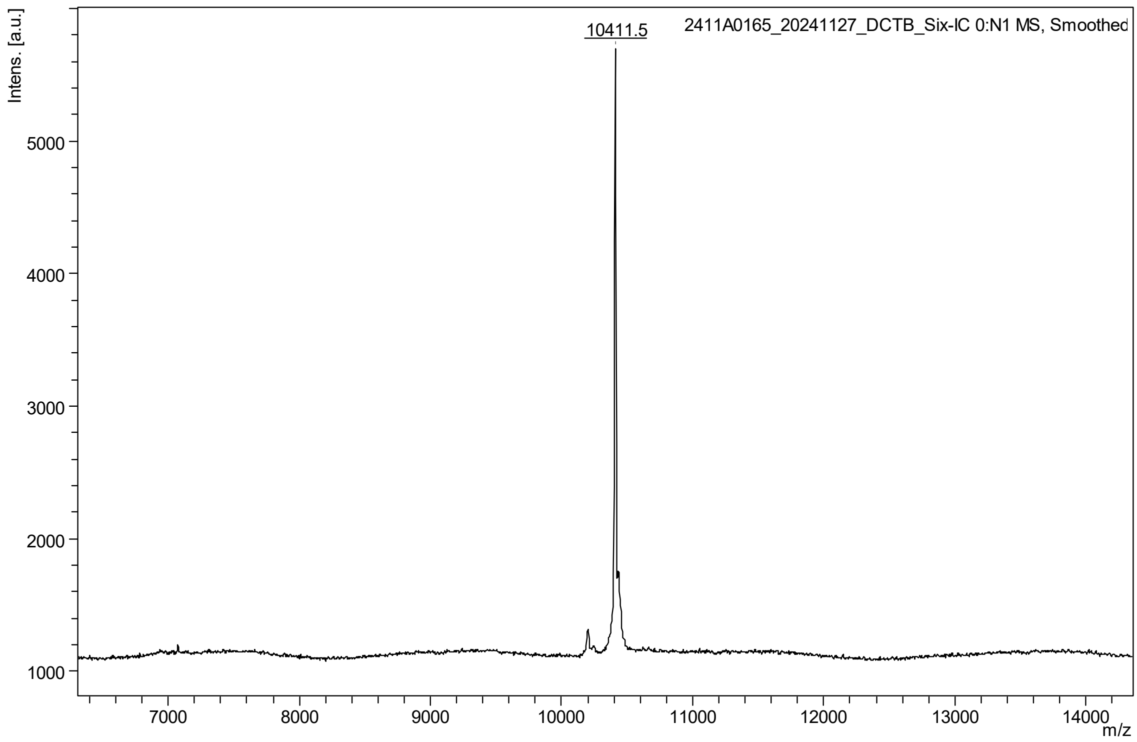

Supplementary Fig. 9. The mass spectrum of **Six-IC**.

High temperature GPC Measurement

The molecular weights of Six-IC was obtained on an Acquity Advanced Polymer Chromatography (Waters) with a high-temperature chromatograph in 1,2,4-trichlorobenzene at 150 °C and using a calibration curve of polystyrene standards.

Workbook Details
Eluent: TCB stabilised with 0.0125% BHT
Column Set: PLgel MIXED-B LS 300x7.5mm x2
Detector: RI
Flow Rate: 1.00 ml/min
Temperature: 150
Injection Volume: 200.0 ul

Analysis Using Method: 20240901
Comments:

Calibration Used: 2024/9/7 10:39:10
High Limit MW RT: 10.95 mins
High Limit MW: 6217712
K: 17.5000
Low Limit MW RT: 17.27 mins
Low Limit MW: 623
Alpha: 0.6700

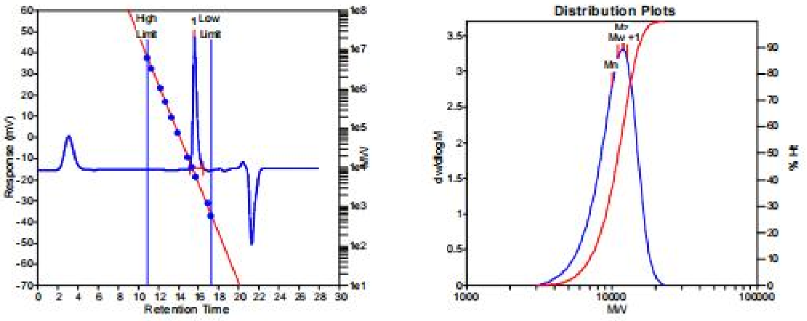

MW Averages

Peak No	Mp	Mn	Mw	Mz	Mz+1	Mv	PD
1	11942	10001	10974	11844	12624	10792	1.09729

Processed Peaks

Peak No	Name	Start RT (mins)	Max RT (mins)	End RT (mins)	Pk Height (mV)	% Height	Area (mV.secs)	% Area
1		15.18	15.60	16.50	61.8949	100	1700.27	100

Supplementary Fig. 10. High-temperature GPC curve of Six-IC.

Photovoltaic Performance of GMA-based Binary OSCs.

Supplementary Table 1. Summary of molecular weight and photovoltaic parameters for high-performance GMA-based binary OSCs.

Donor	Acceptor	M	J_{SC} [mA cm^{-2}]	V_{OC} [V]	FF [%]	PCE [%]	Overall yield [%]	References
PM6	dBTIC γ-EH	2757.9	21.43	0.92	73.28	14.48	18.48	*Angew. Chem. Int. Ed.* **2022**, *61*, e202201844.
PBQx-H-TF	dBTIC γ-BO	3094.6	23.41	0.91	75.49	16.06	14.96	

PM6	2BTP-2F-T	3473.1	25.50	0.911	78.28	18.19	29.34	*Adv. Sci.* **2022**, *9*, e2202513.
PM6	QM1	3585.3	24.37	0.837	71.79	17.05	22.05	*Sci. China Chem.* **2022**, *65*, 1374.
PBDB-T	OY2	3977.7	24.37	0.837	71.79	14.82	19.26	
PBDB-T	OY3	5968.8	23.76	0.839	74.58	15.05	20.12	*Nat. Energy* **2022**, *7*, 1180.
PBDB-T	OY4	7959.8	24.39	0.814	74.69	14.97	26.14	
PM6	CH8	3821.7	19.70	0.889	53.5	9.37	16.74	*Chin. J. Polym. Sci.* **2022**, *40*, 921–927.
D18-Cl-B	4A-DFIC	3204.5	22.47	0.905	77.4	15.76	20.52	*Angew. Chem. Int. Ed.* **2022**, *61*, e202207762.
PM6	dT9TBO	3118.6	9.49	0.99	62.09	5.84	13.57	*Angew. Chem. Int. Ed.* **2023**, *62*, e202303066.
PM6	CH-D1	3893.6	23.90	0.949	73.20	16.62	5.20	*Energy Mater.* **2023**, *13*, 2300301.
D18	DYF-TF	3122.2	25.82	0.939	75.30	18.26	19.98	*CCS Chemistry*, **2023**, *5*, 2576.
PM6	DIBP3F-Se	3219.3	25.92	0.917	76.1	18.09	14.10	*Angew. Chem. Int. Ed.* **2023**, *62*, e202302888.
PM6	Tri-Y6-OD	5382.0	25.30	0.916	77.8	18.03	8.40	*Angew. Chem. Int. Ed.* **2023**, *62*, e202308595.
PM6	DYV	3651.2	25.64	0.93	78	18.60	14.00	*ACS Energy Lett.* **2023**, *8*, 1344.
PM6	DYBO	4270.9	24.62	0.968	75.8	18.08	19.60	*Joule* **2023**, *7*, 416.
PM6	DY2	3010.0	26.60	0.87	76.85	17.85	24.92	*Adv. Mater.* **2023**, *35*, 2206563.
PM6	TDY-α	3184.2	26.9	0.864	78.0	18.1	28.26	*Nat. Commun.* **2023**, *14*, 2926.
PM1	DT19	3288.6	23.9	0.867	71.4	14.80	13.43	*Adv. Mater.* **2023**, *36*, 2302592.
PM6	EV-i	3417.0	26.60	0.897	76.56	18.27	27.3	*Angew. Chem. Int. Ed.* **2023**, *62*, e202303551.
PM6	CH8-1	4005.8	24.89	0.923	74.2	17.05	29.2	*Energy Environ. Sci.* **2023**, *16*, 1773.
PM6	CH8-4	4137.4	26.05	0.894	75.5	17.58	17.13	*Angew. Chem. Int. Ed.* **2023**, *62*, e202307962
PM6	G-Trimer	5161.5	26.75	0.896	79.30	19.01	16.39	*Joule.* **2023**, *7*, *2386-2401.*
PM6	TBT	5582.3	24.63	0.94	78.07	18.04	11.20	*Macromolecules.* **2023**, *56*, *8623-8631.*

D18	DYA-I	3625.1	25.67	0.938	78	18.83	22.4	*Adv. Energy Mater.* **2023**, *13*, *2301283*
PM6	DYSe-1	3754.1	27.51	0.885	76.6	18.56	23.46	*Adv. Energy Mater.* **2024**, *14, 2400938.*
PM6	Dimer-2CF	3700.8	26.39	0.900	80.03	19.02	27.51	*Adv. Mater.* **2024**, *36*, 2310046.
PM6	GT-I	5466.2	25.61	0.960	77.42	19.03	4.85	*Adv. Funct. Mater.* **2024,** 2410092
PM6	BTY	5398.8	27.06	0.864	78.01	18.24	21.31	*Angew. Chem. Int. Ed.* **2024**, *63*, *202400590.*
**D18**	**Six-IC**	**10413**	**0.92**	**27.0**	**78.5**	**19.4**	**58.39**	**This work**

UV−Vis Absorption Spectra

For the solution absorption test, polymer donors were dissolved in chloroform (CF) at a concentration of approximately 10^{-5} mol/L. Solid films (approximately 100 nm thick) for the spectrum test were prepared by spin-coating a CF solution of the polymer donor or SMA onto a quartz plate.

UPS and IIPES Measurements

The power setting of 30 nW and a power number of 0.5 were used for UPS measurement. Samples for UPS measurement were 50 nm prepared on glass substrate. The ITO samples coated with ~50 nm films of D18, DTC8, and Six-IC were used for IPES measurements.

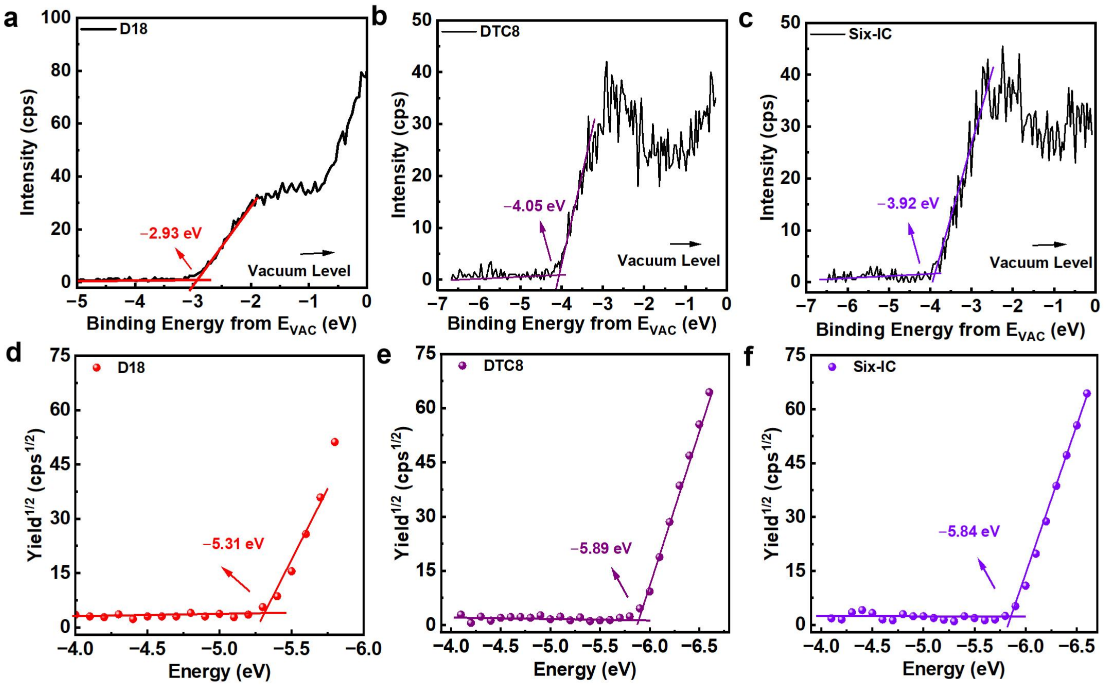

Supplementary Fig. 11. The derived LUMO/HOMO energy levels by (**a-c**) IPES and (**d-f**) UPS measurements.

Thermal Analyses

Thermogravimetric (TG) measurements were conducted using a NETZSCH TG209F3 apparatus, with a heating rate of 10 °C/min under a nitrogen atmosphere. Differential scanning calorimetry (DSC) analysis was conducted using a NETZSCH DSC200F3 apparatus, with a heating and cooling rate of 10 °C/min under a nitrogen atmosphere.

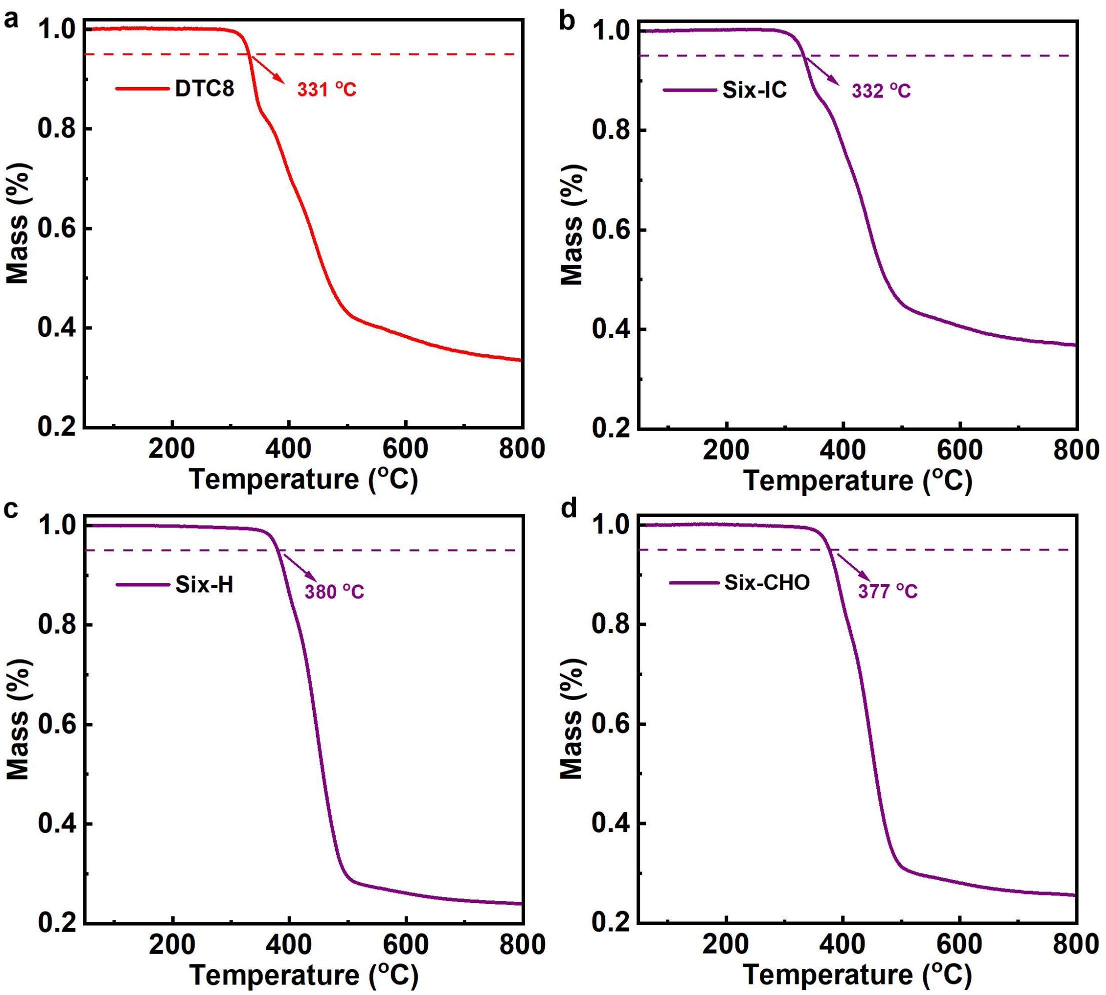

Supplementary Fig. 12. Thermogravimetric analysis (TGA) curves for (**a**) DTC8, (**b**) Six-IC, and the key intermediates (**c**) Six-H and (**d**) Six-CHO.

Computational Analyses

Gaussian 16 (Revision C.02) code [1] was used to perform density functional theory (DFT) calculations at the non-empirically tuned B3LYP-D3(BJ)/TZVP [2-4] level of theory. In the calculation of highest occupied molecular orbital (HOMO) and lowest unoccupied molecular orbital (LUMO) energy levels, the single point energy was calculated under B3LYP-D3(BJ)/Def2TZVP [2-4] level for high precision calculations. We considered a total of 20 excited states from S_1 to S_{20} at the theoretical level of B97X-D/Def2tzvp and calculated the electron-hole distribution and transition dipole moment (TDM). The wavefunction software Multiwfn [5] and VMD [6] were used for analysing Electrostatic Potential (ESP) and electron-hole distribution.

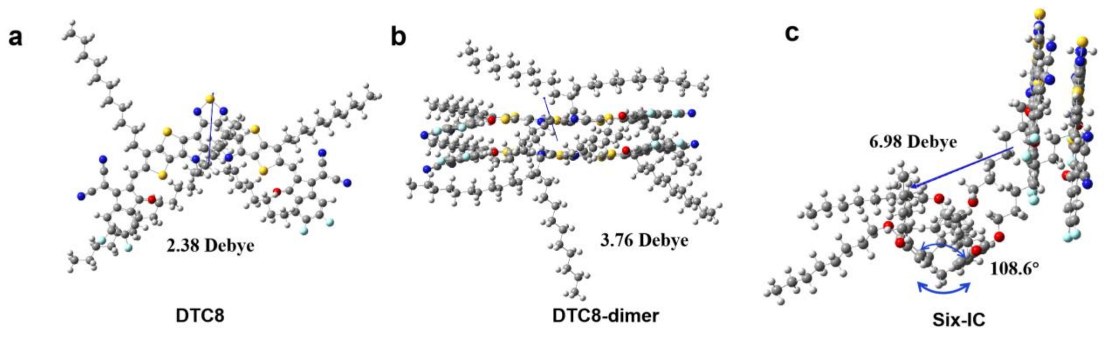

Supplementary Fig. 13. The simulated results of (**a**) DTC8, (**b**) DTC8-dimer, and (**c**) Six-IC model.

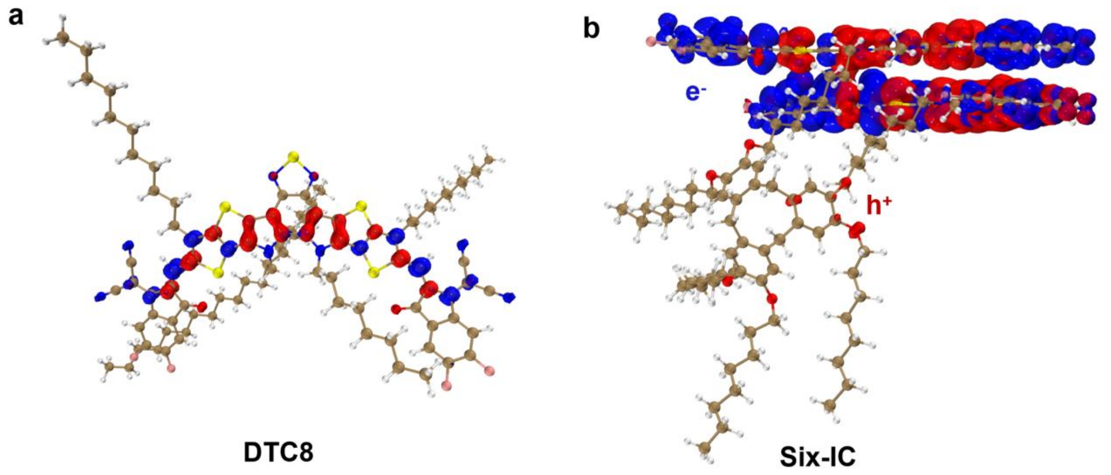

Supplementary Fig. 14. Electron-hole distributions of (**a**) DTC8 and (**b**) Six-IC model.

Fabrication and Characterization of SCLC Devices

The charge carrier mobilities of pristine and blend films are estimated from space-charge-limited current (SCLC) method. The hole-only and electron-only devices were fabricated with the architectures of ITO/PEDOT:PSS/Active layer/MoO_3/Ag and ITO/ZnO/Active layer/PFNDIT-F3N/Ag. Hole-only and electron-only devices were recorded with a Keithley 236 source meter under dark. The hole and electron mobilities were determined by fitting the dark current to the model of single-carrier SCLC, which is described by the equation:

$$J = \frac{9}{8}\varepsilon_0\varepsilon_\gamma\mu\frac{V^2}{d^3} \qquad (1)$$

where J is the current density, μ is the zero-field mobility, ε_0 is the permittivity of free space, ε_r is the relative permittivity of the material, d is the thickness of the active layers, and V is the effective voltage. The effective voltage was obtained by subtracting the built-in voltage (V_{bi}) and the voltage drop (V_s) from the series resistance of the whole device except for the active layers from the applied voltage (V_{appl}), $V = V_{appl} - V_{bi} - V_s$. ($V_{bi} = 0$ and $V_s = 10\times I$, where the value 10 is the resistance of MoO_3 and I is the current of the devices in this work). The hole and electron mobilities can be calculated from the slope of the $J^{1/2}$-V curves.

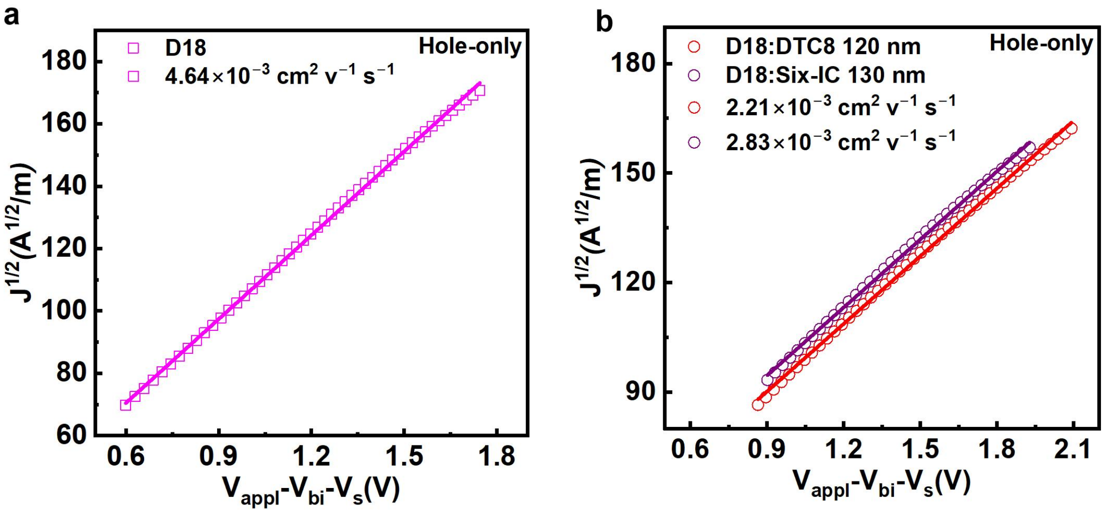

Supplementary Fig. 15. SCLC plots of the (**a**) hole-only and (**b**) electron-only devices. The experimental data are fitted using the SCLC model (solid lines).

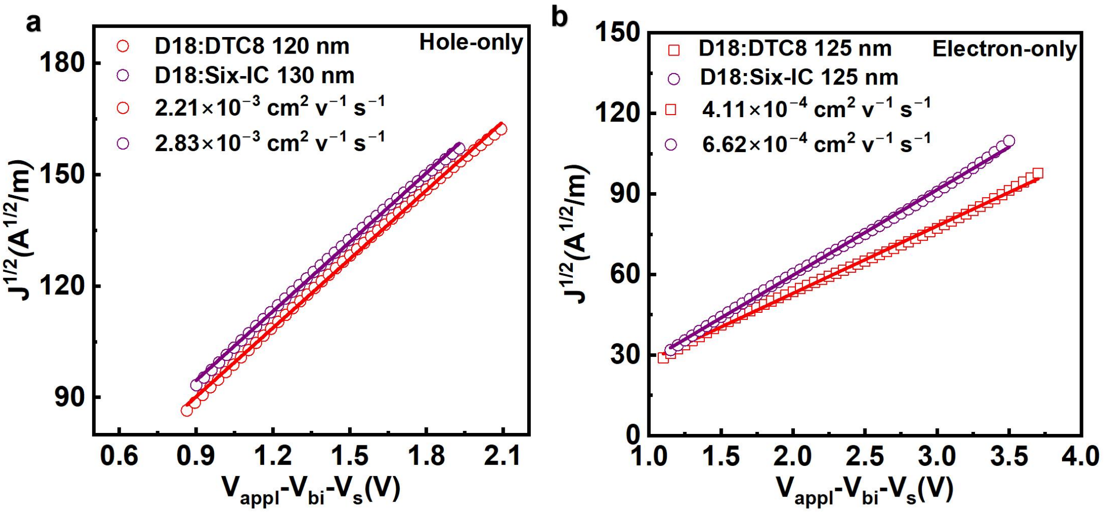

Supplementary Fig. 16. SCLC plots of the (**a**) hole-only and (**b**) electron-only

devices. The experimental data are fitted using the SCLC model (solid lines).

Transient Photovoltage (TPV) Measurement

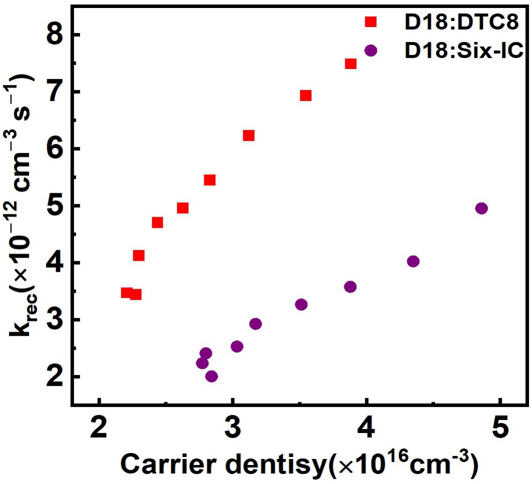

Supplementary Fig. 17. The recombination rate constant (k_{rec}) versus carrier densities (n) curves.

Energy Loss Measurements

The electroluminescence spectra were acquired by a high-sensitivity spectrometer (QE Pro, Ocean Optics), while the external quantum efficiency of EL was determined by measuring the emitted photons in all directions through an integrated sphere by using a calibrated spectrometer (QE Pro, Ocean Optics), with the device injected by an external current/voltage source with constant current density.

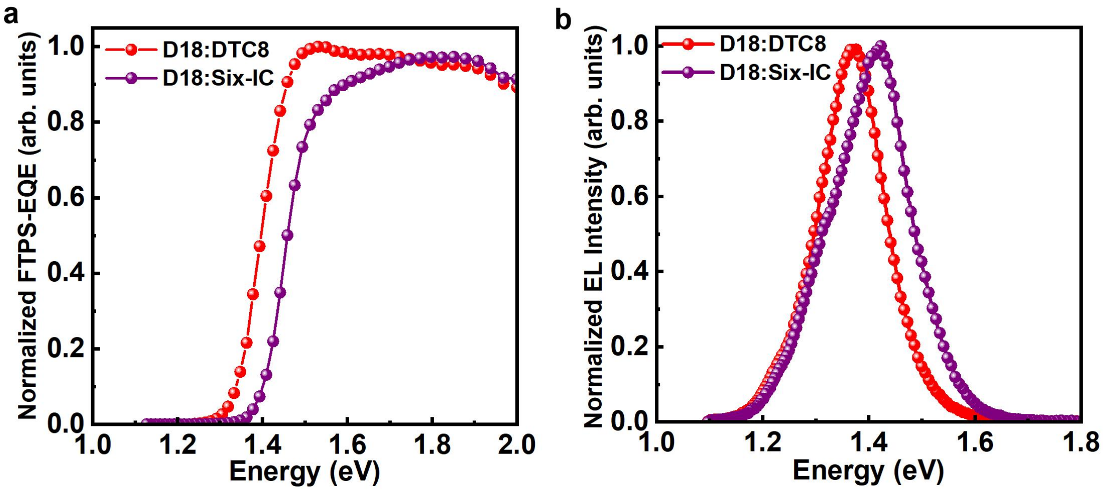

Supplementary Fig. 18.The normalized (**a**) FTPS-EQE and (**b**) EQE_{EL} spectra.

Supplementary Table 2. Detailed energy losses of the related OSCs.

Devices	E_g [eV]	qVoc^{SQ} [eV]	qV_{OC}^{rad} [eV]	E_{loss} [eV]	ΔE_1 [eV]	ΔE_2 [eV]	ΔE_3 [eV]	q$V_{OC}^{Cal.}$ [eV]
D18:DTC8	1.43	1.16	1.07	0.57	0.27	0.09	0.21	0.86
D18:Six-IC	1.48	1.21	1.12	0.56	0.27	0.09	0.20	0.92

Mechanical Properties Measurement

Film Preparation:

For nanoindentation samples, the films of approximately 1 μm thickness were obtained by directly drip-coating the concentrated CF solutions (pure donor, pure acceptor, or donor-acceptor blend) onto glass substrates and annealed at 110°C for 10 minutes.

Mechanical Properties Characterizations:

The nanoindentation tests were conducted using a nanoindenter (TI980, Bruker, USA) equipped with a Berkovich diamond indenter with a radius of approximately 120 nm. To get the values of h_e, h_p, and h_c, a loading rate of 6 μN s^{-1}, a load of 30 μN that held for 2 s, and an unloading rate of 6 μN s^{-1} were preset. Modulus and hardness profiles were recorded in an automatic measurement mode, in which the loading duration was 5 s, holding duration was 2 s, and unloading durations was 5 s. The contact angles were estimated with a goniometer equipped with a CCD camera (Model JY–82, China).

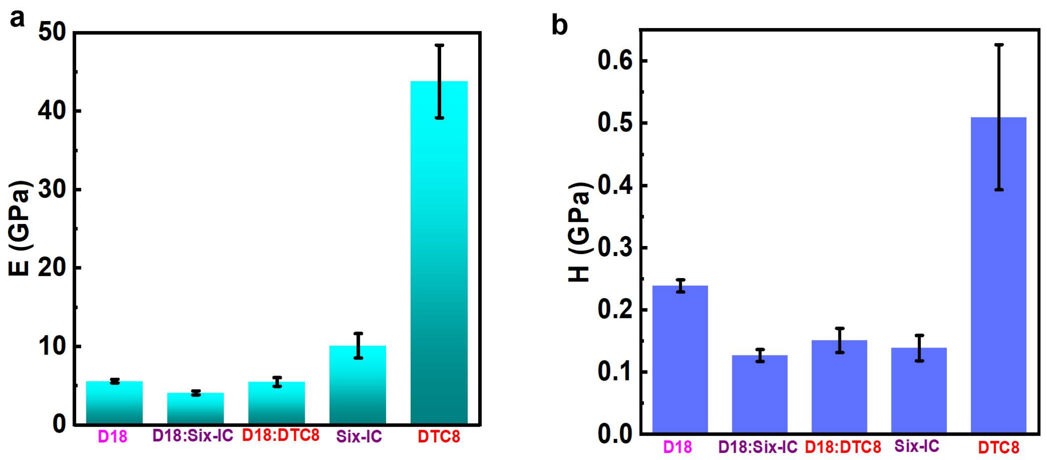

Supplementary Fig. 19. The derived (**a**) modulus and (**b**) hardness from nanoindentation tests

Supplementary Table 3. The modulus and hardness data.

Materials	Average Modulus [GPa]	Average Hardness [GPa]
D18	5.58±0.23	0.239±0.01
DTC8	43.79±4.63	0.51±0.17
Six-IC	43.79±4.63	0.51±0.17
D18:DTC8	43.79±4.63	0.51±0.17
D18:Six-IC	4.09±0.25	0.127±0.01

TEM Measurements

The samples for the TEM measurements were prepared as follows: the active-layer films were spin-cast onto ITO/PEDOT:PSS substrates, and the substrates with the active layers were submerged in deionized water to make the active layers float on the air-water interface. Then, the floated films were picked up on unsupported 200 mesh copper grids for the TEM measurements.

Contact Angles and Surface Energy Parameters

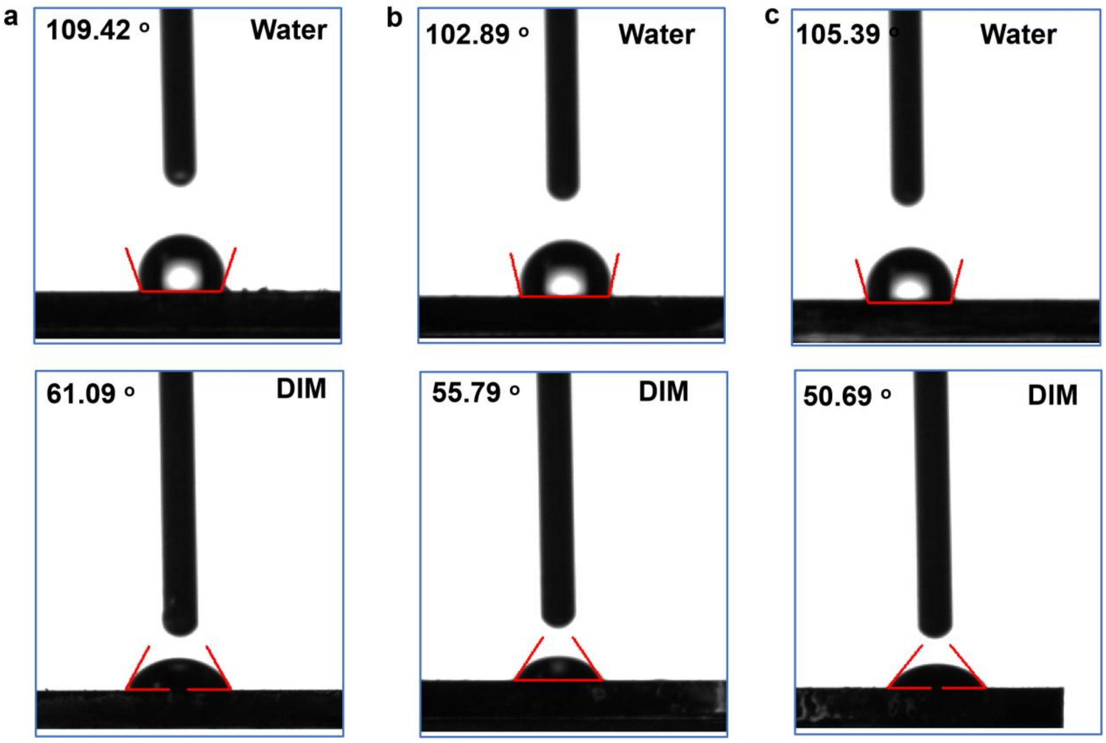

Supplementary Fig. 20. Contact angle images of pristine (**a**) D18, (**b**) DTC8, and (**c**) Six-IC film with water and glycol droplets on top.

Supplementary Table 4. The contact angles and surface energy parameters of the polymer films.

Materials	Contact angle		γ_d [b] [mN m^{-1}]	γ_p [b] [mN m^{-1}]	γ [mN m^{-1}]	$\chi_{donor\text{-}acceptor}$ [c] [K]
	θ_{water} [°]	θ_{oil} [°] [a]				
D18	109.42	61.09	24.63	0.02	24.65	—
DTC8	102.89	55.79	31.79	0.07	31.86	0.46
Six-IC	105.39	50.69	29.79	0.03	29.82	0.25

[a)] θ_{oil} represents the contact angle of glycol;

[b)] γ_d and γ_p represent the surface free energies generated from the dispersion forces and the polar forces, respectively;

[c)] The Flory−Huggins interaction parameters ($\chi_{donor\text{-}acceptor}$) were calculated using the empirical relation: $\chi_{donor\text{-}acceptor}=K(\gamma_{donor}^{1/2}-\gamma_{acceptor}^{1/2})^2$ (K is a constant).

2D GIWAXS measurements

1D GIWAXS patterns was corrected to represent real q_r and q_{xy} axes with the consideration of missing wedge. The critical incident angle was determined by the

maximised scattering intensity from sample scattering with negligible contribution from underneath layer scattering. The shallow incident angle scattering was collected at 0.2°, which renders the incident X-ray as an evanescent wave along the top surface of thin films. The samples for GIWAXS test were prepared by casting solution onto silicon wafer substrates (ca. 15 mm×15 mm), and the active layers were prepared using exactly the same concentration and same procedures as those for device processing.

Supplementary Table 5. GIWAXS parameters of the related D18, DTC8, and Six-IC neat films in IP direction.

Sample	IP (100)			
	q [$Å^{-1}$]	*d*-spacing [Å]	FWHM [$Å^{-1}$]	CCL [Å]
D18	0.299	20.990	0.094	60.190
DTC8	0.258	24.313	0.059	95.960
Six-IC	0.271	23.173	0.091	62.110

Supplementary Table 6. GIWAXS parameters of DTC8, and Six-IC neat films in OOP direction

Sample	OOP (010)			
	q [$Å^{-1}$]	*d*-spacing [Å]	FWHM [$Å^{-1}$]	CCL [Å]
DTC8	1.721	3.649	0.211	26.787
Six-IC	1.565	4.013	0.464	12.181

Supplementary Table 7. GIWAXS parameters of the related D18:DTC8, and D18:Six-IC films in IP direction.

Sample	IP (100)			
	q [$Å^{-1}$]	*d*-spacing [Å]	FWHM [$Å^{-1}$]	CCL [Å]
D18:DTC8	0.291	21.547	0.0830	68.096
D18:Six-IC	0.282	22.270	0.086	65.721

Supplementary Table 8. GIWAXS parameters of D18:DTC8, and D18:Six-IC blend films in OOP direction

Sample	OOP (010)			
	q [Å^{-1}]	d-spacing [Å]	FWHM [Å^{-1}]	CCL [Å]
D18:DTC8	1.716	3.660	0.197	28.690
D18:Six-IC	1.604	3.92	0.247	22.883

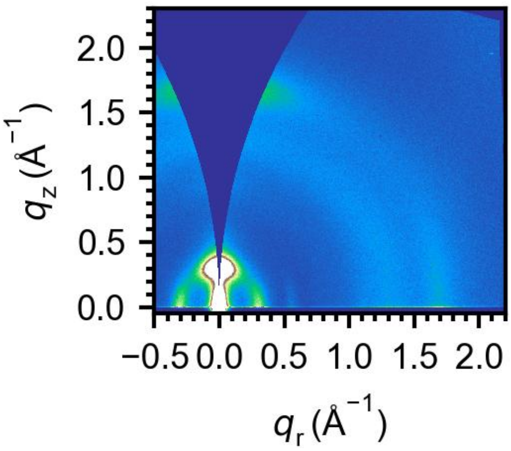

Supplementary Fig. 21. 2D-GIWAXS patterns of D18 pure film.

Ultra-fast TAS Measurements

Transient absorption measurement was conducted on a commercial pump-probe femtosecond transient absorption (TA) spectrometer Helios (Ultrafast System, USA). Ultrafast laser pulses (800 nm, < 35 fs pulse duration, 7 W) was generated by 1 kHz Ti:Sapphire regenerative amplifier (Astrella, Coherent, USA). 40% of the fundamental pulses (7 W) was used to pump the commercial collinear optical parametric amplifier (TOPAS Prime, Light-Conversion, Lithuania) for generating tunable wavelength pump pulse to 800 nm. The pump beam is chopped at 500 Hz. 15% of the fundamental pulses was routed onto a mechanical delay stage (within 7 ns) and passed through a sapphire crystal to generate supercontinuum probe light (450-750 nm). The pump light and probe light were focused on a same spot (2 mm diameter) of the thin films placed on a quartz. Data analysis is performed by Surface Xplorer software. The incident power is measured with a calibrated laser power meter (Newport).

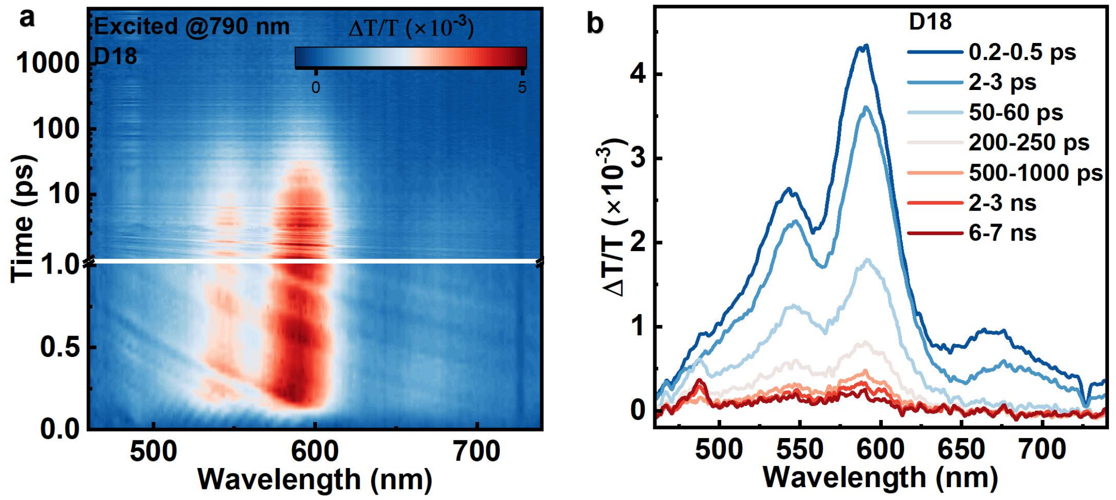

Supplementary Fig. 22. (**a**) 2D contour map of TA signals recorded from D18. (**b**) TA spectra of D18 pure film at different delay times.

Photoinduced Force Microscopy (PiFM) Measurement

Nanoscale chemical imaging and infrared spectroscopy were conducted using an AFM-IR nano IR3 system (Bruker, USA) equipped with a quantum cascade laser (QCL). For nanoscale chemical imaging, the amplitude corresponding to specific laser wavelengths was utilized. Nanoscale IR spectra were obtained by measuring the amplitude of cantilever oscillations within the range of 800 to 1800 cm^{-1}, with a resolution of 2 cm^{-1}. This was achieved using a gold-coated silicon cantilever featuring a nominal spring constant of 0.3 N m^{-1}.

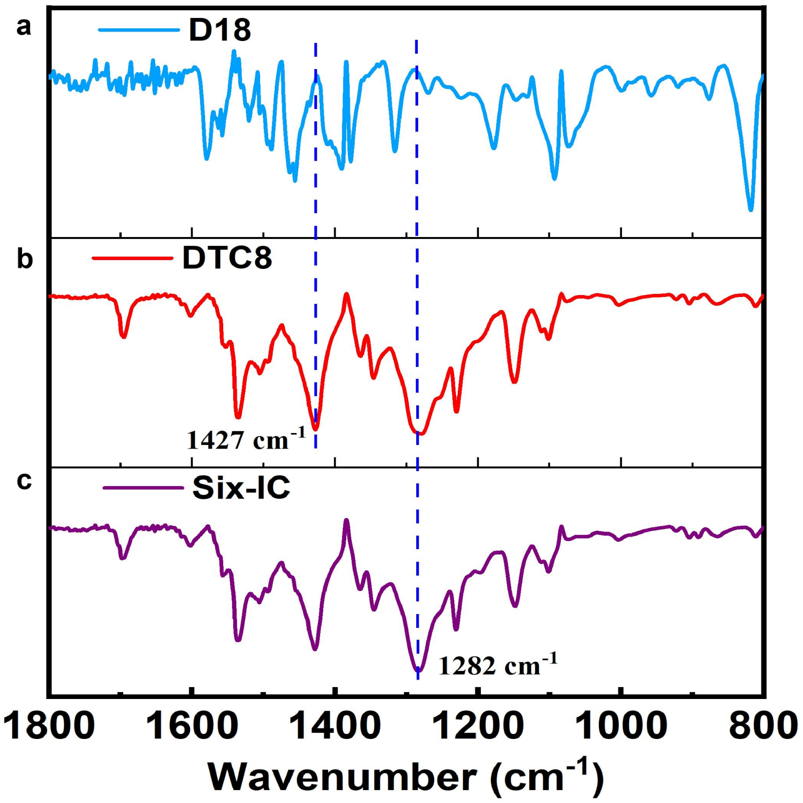

Supplementary Fig. 23. The characteristic FT-IR peaks of (**a**) D18, (**b**) DTC8, and (**c**) Six-IC.

Stability Measurement

The testing devices were fabricated under the same preparation conditions as those used for the *J-V* curve measurements. These samples were then transferred to a nitrogen-filled glovebox, maintaining the same controlled water-oxygen environment. The devices were placed on a thermal-conducting copper plate, with insulating glass pieces inserted between the copper plate and the device. The temperature of the copper plate was controlled to either room temperature or 85°C via heat conduction through the glass sheet, providing a stable thermal environment for the device measurements. Before the data collection, the devices was pre-aged for 72 hours in the room temperature or 85°C. The *J-V* characteristics of the devices were regularly checked, and the photovoltaic parameters were automatically calculated based on the resulting J-V curves.

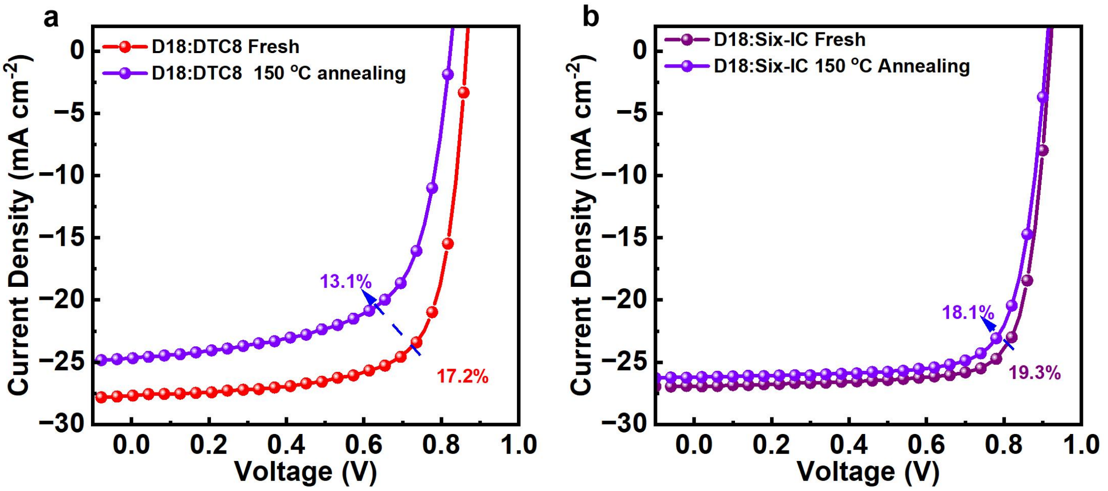

Supplementary Fig. 24. The *J-V* curves of (**a**) D18:DTC8 and (**b**) D18:Six-IC before and after annealing at 150 °C for 3 hours.

Supplementary References

1. Gaussian 16, Revision C.02, M. J. Frisch, G. W. Trucks, H. B. Schlegel, G. E. Scuseria, M. A. Robb, J. R. Cheeseman, G. Scalmani, V. Barone, G. A. Petersson, H. Nakatsuji, X. Li, M. Caricato, A. V. Marenich, J. Bloino, B. G. Janesko, R. Gomperts, B. Mennucci, H. P. Hratchian, J. V. Ortiz, A. F. Izmaylov, J. L. Sonnenberg, D. Williams-Young, F. Ding, F. Lipparini, F. Egidi, J. Goings, B. Peng, A. Petrone, T. Henderson, D. Ranasinghe, V. G. Zakrzewski, J. Gao, N. Rega, G. Zheng, W. Liang, M. Hada, M. Ehara, K. Toyota, R. Fukuda, J. Hasegawa, M. Ishida, T. Nakajima, Y. Honda, O. Kitao, H. Nakai, T. Vreven, K. Throssell, J. A. Montgomery, Jr., J. E. Peralta, F. Ogliaro, M. J. Bearpark, J. J. Heyd, E. N. Brothers, K. N. Kudin, V. N. Staroverov, T. A. Keith, R. Kobayashi, J. Normand, K. Raghavachari, A. P. Rendell, J. C. Burant, S. S. Iyengar, J. Tomasi, M. Cossi, J. M. Millam, M. Klene, C. Adamo, R. Cammi, J. W. Ochterski, R. L. Martin, K. Morokuma, O. Farkas, J. B. Foresman, and D. J. Fox, Gaussian, Inc., Wallingford CT, 2019. D. J. Fox, Gaussian 16, Revision C.02, Gaussian, Inc., Wallingford CT, (2019).
2. Becke AD. A new mixing of Hartree–Fock and local density-functional theories. *J.Chem. Phys.* **98**, 1372-1377 (1993).
3. Grimme S, Antony J, Ehrlich S, Krieg H. A consistent and accurate ab initio

parametrization of density functional dispersion correction (DFT-D) for the 94 elements H-Pu. *J. Chem. Phys.* **132**, 154104 (2010).

4. Pritchard BP, Altarawy D, Didier B, Gibson TD, Windus TL. New Basis Set Exchange: An Open, Up-to-Date Resource for the Molecular Sciences Community. *J. Chem. Inf. Model.* **59**, 4814-4820 (2019).
5. Lu T, Chen F. Multiwfn: A multifunctional wavefunction analyzer. *J. Comput. Chem.* **33**, 580-592 (2012).
6. Humphrey W, Dalke A, Schulten K. VMD: Visual molecular dynamics. *J. Mol. Graph.* **14**, 33-38 (1996).
