# Supplementary material for: A dendritic hexamer acceptor enables 19.4% efficiency with exceptional stability in organic solar cells
Source: Nat Commun. 2025 Jan 20;16:871. doi: 10.1038/s41467-025-56225-x (PMC11747272; doi:10.1038/s41467-025-56225-x)
Supplement: Supplementary file 2 — Description Of Additional Supplementary File [file 41467_2025_56225_MOESM2_ESM.pdf]

Description of Additional supplementary files

Supplementary data 1: Atomic coordinates of DTC8 for DFT calculations

Supplementary data 2: Atomic coordinates of Six-IC for DFT calculations

Supplementary data 3: Final configuration of DTC8 for MD simulations

Supplementary data 4: Final configuration of Six-IC for MD simulations

Supplementary data 5: Initial configuration of DTC8 for MD simulations

Supplementary data 6: Initial configuration of Six-IC for MD simulations
